# Subtotal gastrectomy for gastric tube cancer using intraoperative indocyanine green fluorescence method

**DOI:** 10.1016/j.ijscr.2020.04.049

**Published:** 2020-05-11

**Authors:** Ippei Yamana, Takuo Murakami, Shintaro Ryu, Jun Ichikawa, Yuki Shin, Nobuhiko Koreeda, Hiroto Sannomiya, Keisuke Sato, Tatsuya Okamoto, Yasuo Sakamoto, Yasushi Yoshida, Jun Yanagisawa, Tomoaki Noritomi, Suguru Hasegawa

**Affiliations:** a Department of Surgery, Fukuoka Tokushukai Hospital, 4-5 Sukukita, Kasuga, Fukuoka, 816-0864, Japan; b Department of Gastroenterological Surgery, Fukuoka University School of Medicine, 7-45-1, Nanakuma, Jonan-ku, Fukuoka, 814-0180, Japan

**Keywords:** Gastric tube cancer, Indocyanine green fluorescence, Esophagectomy

## Abstract

**Introduction:**

Currently, the frequency of evaluating the flow of a reconstructed gastric tube using indocyanine green (ICG) fluorescence has been increasing. However, it has been difficult to decide on the operation method for patients with gastric tube cancer (GTC). We herein report a case in which ICG was effective in a patient with resection of GTC.

**Presentation of case:**

An 83-year-old man underwent subtotal esophagectomy with gastric tube reconstruction via the retrosternal route for esophageal cancer and right hemicolectomy for ascending colon cancer 16 years earlier. Postoperatively, the proximal part of the gastric tube had poor blood flow. Therefore, the patient underwent proximal-side resection of the gastric tube. Thereafter, free jejunal graft reconstruction was performed. The patient had not developed recurrence at that point. Recently, the patient visited the hospital complaining of nausea and chest discomfort. Upper gastrointestinal endoscopy revealed a type 0-IIa + IIc lesion located around the pylorus. A biopsy showed adenocarcinoma. Based on these findings, the patient was diagnosed with gastric tube cancer (cT1bN0M0StageI). The invasion depth of the cancer was predicted to be widespread submucosal invasion. Therefore, the patient underwent surgery. Intraoperatively, we evaluated the flow of the gastric tube after clamping the right gastroepiploic artery using ICG fluorescence. As a result, the flow of the gastric tube was deemed insufficient. Consequently, subtotal gastrectomy was performed with preservation of the right gastroepiploic artery via Roux-en-Y reconstruction.

**Discussion:**

ICG fluorescence is useful for evaluating the flow of the gastric tube helping to decide the operating method.

**Conclusion:**

We herein report a case of subtotal gastrectomy for GTC using intraoperative ICG fluorescence.

## Highlights

• We presented a patient with gastric tube cancer who successfully underwent subtotal gastrectomy with intraoperative ICG fluorescence.
• ICG fluorescence is useful for evaluating the flow of the gastric tube and helping to determine the operating method.

## 1 Introduction

Metachronous second cancer is a possibility after esophagectomy for esophageal cancer. Most such cases involve pharynx cancer, with gastric tube cancer (GTC) the second-most frequent cause. The estimated 10-year cumulative rate of GTC after esophagectomy is 5.7%–8.1% [1,2].

The prognosis of esophageal cancer has been improved thanks to multimodal therapy, including chemotherapy, radiation therapy, and photodynamic therapy. However, the frequency of GTC after esophagectomy is expected to increase in the future. Endoscopic resection is an appropriate method for treating early-stage GTC. However, in cases of advanced GTC, patients require surgical resection. In cases of radical resection, total gastric tube resection is better than subtotal gastrectomy for ensuring sufficient lymph node dissection. However, total gastric tube resection is a particularly invasive surgery, especially for elderly patients.

Publications concerning the utility of indocyanine green (ICG) fluorescence for gastrointestinal cancer surgeries have been increasing in frequency [[3], [4], [5]]. We herein report a case of GTC managed using intraoperative ICG fluorescence with a literaturereview in line with the SCARE criteria [6].

## 2 Presentation of case

**Fig. 1:**
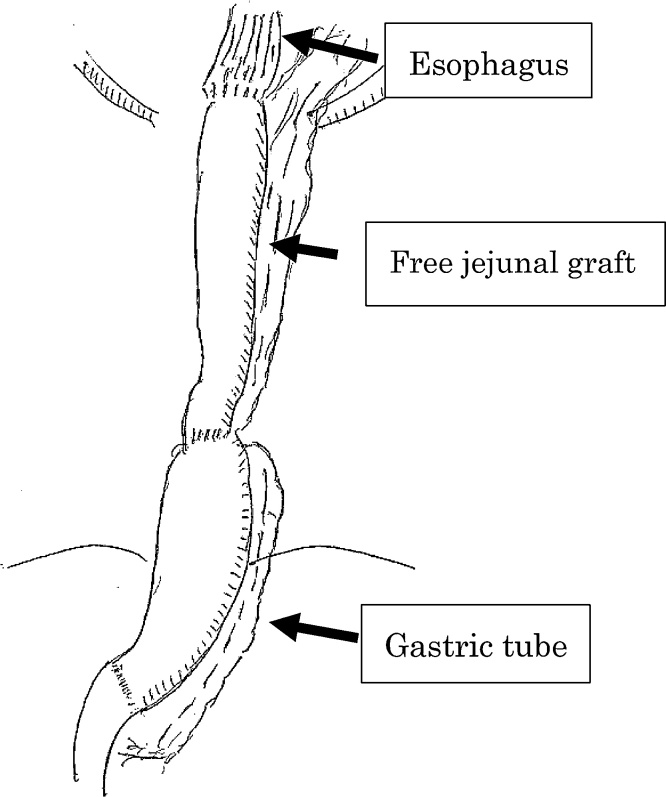
A schematic illustration of the reconstructed status.

Sixteen years ago, the present patient underwent subtotal esophagectomy with gastric tube reconstruction via the retrosternal route for esophageal squamous cell carcinoma and right hemicolectomy for ascending colon adenocarcinoma. The initial esophageal cancer stage was pT3N1M0StageIII, and the colon cancer stage was pT2N0M0StageI. Postoperatively, the proximal part of the gastric tube had poor blood flow. Therefore, the patient underwent proximal-side resection of the gastric tube. Thereafter, free jejunal graft reconstruction was performed (Fig. 1). The patient did not develop recurrence subsequently.

**Fig. 2:**
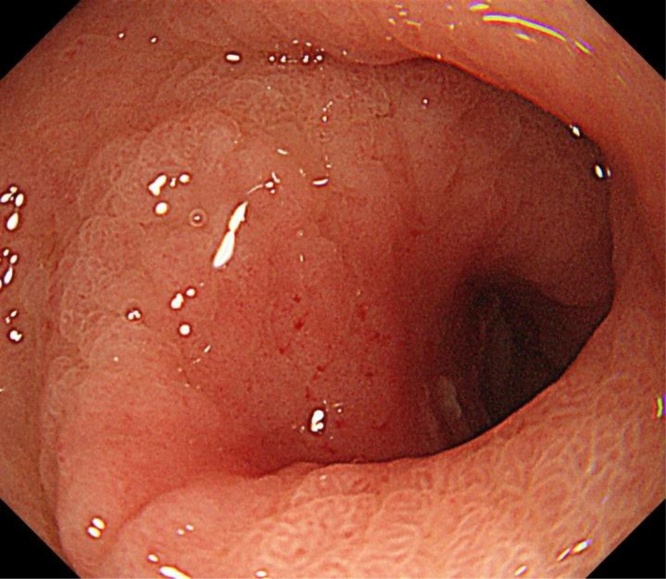
Upper gastrointestinal endoscopy revealed a type 0-IIa + IIc lesion located around the pylorus.

Recently, however, the patient visited the hospital complaining of nausea and chest discomfort. Upper gastrointestinal endoscopy revealed a type 0-IIa + IIc lesion located around the pylorus (Fig. 2). A biopsy showed well-differentiated adenocarcinoma, and GTC was diagnosed. The invasion depth of the cancer was predicted to be submucosal invasion on four fifth round. Computed tomography revealed no swollen lymph nodes or distant metastasis. We considered endoscopic submucosal dissection (ESD) to be difficult from a radical perspective and the risk of postoperative stenosis. Therefore, the patient underwent surgery. Preoperatively, we discussed the ideal approach (total gastric tube resection or otherwise) during a surgical team conference. Total gastric tube resection was considered the best approach as radical therapy, including lymph node dissection of the right gastroepiploic region. However, the operation was deemed too stressful for the elderly patient. It was possibility of developing collateral circulation to the gastric tube from the cranial side. We decided to evaluate the flow of the collateral circulation using ICG fluorescence.

**Fig. 3:**
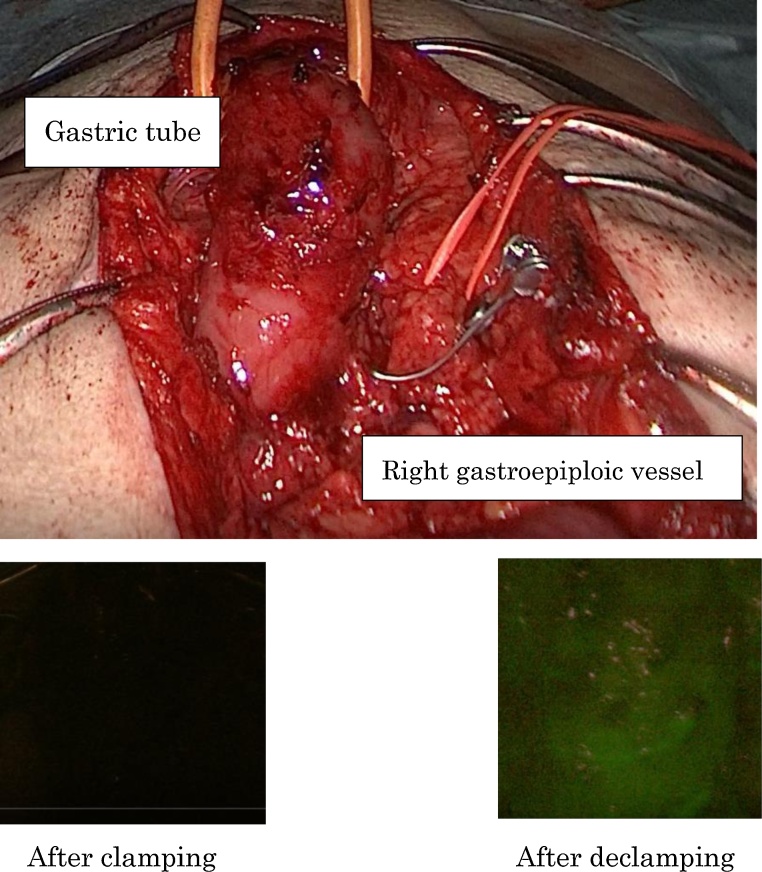
The flow of the gastric tube was considered insufficient after clamping the gastroepiploic artery according to ICG fluorescence.

**Fig. 4:**
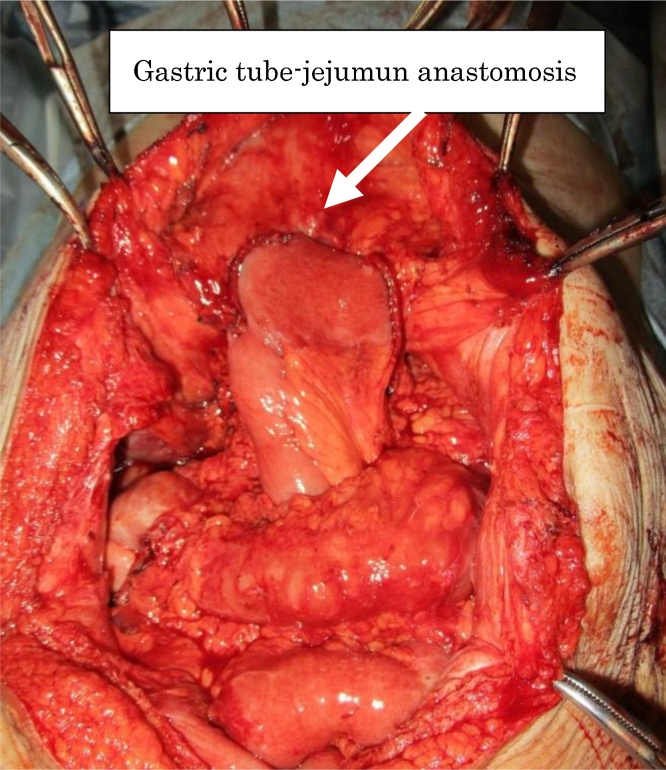
Subtotal gastrectomy with preservation of the right gastroepiploic artery with Roux-en-Y reconstruction was performed.

The patient underwent laparotomy with an upper median incision. The pedicle of the right gastroepiploic artery was identified. Thereafter, we evaluated the flow of the gastric tube after clamping the gastroepiploic artery using ICG fluorescence imaging with an Image 1-S (KARL STORZ, Germany), and video recording. ICG was injected as a 2.5-mg bolus via a peripheral vein. As a result, the flow of gastric tube was deemed to be insufficient (Fig. 3). We therefore considered that the gastric tube was likely not supplied via the collateral circulation. Consequently, subtotal gastrectomy with preservation of the right gastroepiploic artery and Roux-en-Y reconstruction was performed (Fig. 4). The gastric tube-jejunum anastomosis was performed using overlap method. The lymphectomy was not performed. The duration of the operation was 302 min, and the blood loss was 150 mL.

Regarding the pathological findings, the resected specimen was 0-IIa + IIc type, measuring 29 × 54 mm. Well-differentiated adenocarcinoma without lymphovascular invasion was diagnosed. There was no lymph node metastasis, and no carcinoma was present at the surgical margin of the oral or anal side.

Superficial surgical site infection developed after surgery. Oral intake was started at postoperative day (POD) 14. Superficial surgical site infection was occurred on POD7. The patient was discharged on POD 68 and was still doing well nine months after surgery.

## 3 Discussion

This study demonstrated that subtotal gastrectomy with preservation of the proximal region of the gastric tube using ICG fluorescence could be safely performed without serious complications in an elderly patient. ICG fluorescence is a safe and promising method for assessing perfusion. It has been used for lymphography, angiography, and sentinel node dissection and has proven useful for more clearly distinguishing ischemic and non-ischemic areas. This approach also allows for the intraoperative real-time assessment of the blood supply in the gastric tube.

Sakaki et al. suggested that distal gastrectomy, including resection of the right gastroepiploic artery, did not result in insufficient perfusion of the remnant gastric tube [7]. It was possible to reflect retrograde perfusion at the resected distal margin of the right gastroepiploic artery, suggesting that the bidirectional blood supply through the gastroepiploic vessel arcade is a long-term result of collateral vascularization after esophagectomy and gastric pull-up through an intramural vascular network or an arterial network from the omental branches [8]. However, in our case, revascularization in the upper region of the gastric tube was not confirmed using ICG fluorescence intraoperatively. Because the patient had already undergone free jejunal graft reconstruction after esohagectomy with gastric tube reconstruction, we considered that sufficient collateral vascularization was not recognized using ICG fluorescence.

From an oncological perspective, we consider subtotal gastrectomy to be inferior to total gastric tube resection, as total gastric tube resection is radical surgery including a negative lateral margin and dissection of all lymph nodes around the gastric tube. However, total gastric tube resection is associated with several major issues. Severe adhesions around the gastric tube, left recurrent laryngeal nerve palsy, and remnant esophagus injury seem to occur relatively rarely, consequently leading to complications of anastomotic leakage and pulmonary issues. In the present case, subtotal gastrectomy was performed in an elderly patient. Intraoperatively, collateral vascularization was not recognized using ICG fluorescence after clamping the right gastroepiploic artery. We considered subtotal gastrectomy including dissection of the lymph nodes around the pyloric region to be impossible. Therefore, we performed subtotal gastrectomy with preservation of the right gastroepiploic vessels instead.

In conclusion, we presented a patient with GTC who successfully underwent subtotal gastrectomy with intraoperative ICG fluorescence. ICG fluorescence is useful for evaluating the flow of the gastric tube and helping to determine the operating method.

## Declaration of Competing Interest

Conflicts of interest is nothing.

## Sources of funding

Sources of funding is nothing.

## Ethical approval

Ethical approval has been given.

## Consent

A patients in case report was given Informed consent.

## Author contribution

Ippei Yamana (Writing the paper, study concept or design), Takuo Murakami (Study concept or design), Shintaro Ryu (Others), Jun Ichikawa (Others), Yuki Shin (Others), Nobuhiko Koreeda (Others), Hiroto Sannomiya (Others), Keisuke Sato (Others), Tatsuya Okamoto (Others), Yasuo Sakamoto (Others), Yasushi Yoshida (Others), Tomoaki Noritomi (Others), Suguru Hasegawa (Others).

## Registration of research studies

This paper is case report.

## Guarantor

Suguru Hasegawa Professor.

## Provenance and peer review

Not commissioned, externally peer-reviewed.

## References

[1] Bamba T., Kosugi S., Takeuchi M., Kobayashi M., Kanda T., Matsuki A., Hatakeyama K. (2010). Surveillance and treatment for second primary cancer in the gastric tube after radical esophagectomy. Surg. Endosc..

[2] Lee G.D., Kim Y.H., Choi S.H., Kim H.R., Kim D.K., Park S.I. (2014). Gastric conduit cancer after oesophagectomy for oesophageal cancer: incidence and clinical implications. Eur. J. Cardiothorac. Surg..

[3] Kumagai Y., Hatano S., Sobajima J., Ishiguro T., Fukuchi M., Ishibashi K.I., Mochiki E., Nakajima Y., Ishida H. (2018). Indocyanine green fluorescence angiography of the reconstructed gastric tube during esophagectomy: efficacy of the 90-second rule. Dis. Esophagus.

[4] Kitagawa H., Namikawa T., Iwabu J., Fujisawa K., Uemura S., Tsuda S., Hanazaki K. (2018). Assessment of the blood supply using the indocyanine green fluorescence method and postoperative endoscopic evaluation of anastomosis of the gastric tube during esophagectomy. Surg. Endosc..

[5] Van Daele E., Van Nieuwenhove Y., Ceelen W., Vanhove C., Braeckman B.P., Hoorens A., Van Limmen J., Varin O., Van de Putte D., Willaert W., Pattyn P. (2019). Near-infrared fluorescence guided esophageal reconstructive surgery: a systematic review. World J. Gastrointest. Oncol..

[6] Agha R.A., Borrelli M.R., Farwana R., Koshy K., Fowler A., Orgill D.P., For the SCARE Group (2018). The SCARE 2018 statement: updating consensus surgical CAse REport (SCARE) guidelines. Int. J. Surg..

[7] Sakaki A., Kanamori J., Sato A., Okada N., Ishiyama K., Kurita D., Oguma J., Daiko H. (2019). Gastric tube cancer after esophagectomy-retrograde perfusion after proximal resection of right gastroepiploic artery. Int. J. Surg. Case Rep..

[8] Saito T., Yano M., Motoori M., Kishi K., Fujiwara Y., Shingai T., Noura S., Ohue M., Ohigashi H., Ishikawa O. (2012). Subtotal gastrectomy for gastric tube cancer after esophagectomy: a safe procedure preserving the proximal part of gastric tube based on intraoperative ICG blood flow evaluation. J. Surg. Oncol..

